# Supplementary material for: Pretreatment with Betablockers, a Potential Predictor of Adverse Cardiovascular Events in Takotsubo Syndrome
Source: Biomedicines. 2022 Feb 16;10(2):464. doi: 10.3390/biomedicines10020464 (PMC8962389; doi:10.3390/biomedicines10020464)
Supplement: Supplementary file 1 [file biomedicines-10-00464-s001.zip › biomedicines-1567508-supplementary.pdf]

Suppl. Table S1. Univariate and multivariate binary logistic of parameters of clinical presentation, precipitating factors, cardiovascular risk factors and pretreatment with the risk of cardiac decompensation.

	**Cardiac decompensation**			**Cardiac decompensation**		
	**Univariate HR**			**Multivariate HR**		
	B	95 % CI	*p*=	B	95 % CI	*p*=
Age (years)	1,081	1.015-1.151	0,016	1,064	0.995-1.139	0,071
BMI (kg/m^2)	1,052	0.943-1.175	0,212			
Creatinine (mg/dl)	2,588	0.296-22.609	0,390			
CRP (mg/l)	0,998	0.840-1.186	0,983			
(hs)-troponin (pg/ml)	1,002	1.000-1.004	0,036			
Pro-BNP (pg/ml)	1,001	1.000-1.000	0,196			
EF (%)	0,941	0.873-1.014	0,110	0,943	0.866-1.026	0,173
Betablockers (n)	4,278	1.314-13.928	0,016	3,308	0.895-12.229	0,073
ACE/AT-1 inhibitor (n)	3,001	0.968-9.302	0,057			
Anticoagulation (n)	2,000	0.442-9.056	0,368			
Diuretics (n)	3,577	0.863-14.817	0,079			
SSRI/SNRI (n)	2,692	0.756-9.586	0,126			
Inhalation therapy (n)	1,846	0.517-6.597	0,345			

BMI (Body Mass Index), CRP (C Reactive Protein), EF (Ejection fraction), HbA1c (hemoglobin A1c), SSRI/SNRI (Selective Serotonin Reuptake Inhibitor/selective noradrenaline reuptake inhibitor)

Suppl. Table S2. Univariate and multivariate binary logistic of parameters of clinical presentation, precipitating factors, cardiovascular risk factors and pretreatment with the risk of arrythmia.

	**Arrhythmia**			**Arrhythmia**		
	**Univariate HR**			**Multivariate HR**		
	B	95 % CI	*p*=	B	95 % CI	*p*=
Age (years)	0,956	0.895-1.020	0,169	0,944	0.877-1.017	0,128
BMI (kg/m^2)	0,923	0.788-1.081	0,322			
Creatinine (mg/dl)	0,010	0.000-3.149	0,116			
CRP (mg/l)	0,777	0.414-1.459	0,433			
(hs)-troponin (pg/ml)	1,000	0.998-1.002	0,871			
Pro-BNP (pg/ml)	1,000	1.000-1.000	0,667			
EF (%)	0,941	0.857-1.034	0,208	0,941	0.855-1.035	0,941
Betablockers (n)	2,286	0.570-9.173	0,244	2,444	0.493-12.110	0,274
ACEi/AT-1 (n)	0,510	0.117-2.223	0,370			
Anticoagulation (n)	3,514	0.681-18.126	0,133			
Diuretics (n)	0,001	0.001-0.067	0,999			
SSRI/SNRI (n)	0,795	0.147-4.316	0,791			
Inhalation therapy (n)	0,344	0.039-3.045	0,337			

BMI (Body Mass Index), CRP (C Reactive Protein), EF (Ejection fraction), HbA1c (hemoglobin A1c), SSRI/SNRI (Selective Serotonin Reuptake Inhibitor/selective noradrenaline reuptake inhibitor)
